# Unilateral Renal Castleman Disease Presenting with Acute Abdominal Pain: A Case Report and Literature Review

**DOI:** 10.2174/0115734056450501260506045647

**Published:** 2026-05-17

**Authors:** Ying Wang, Hai Chen, Shutong Chen, Kuixin Fan, Qi Dai

**Affiliations:** 1 Department of Radiology, Ningbo No. 2 Hospital, Ningbo, China; 2 School of Medical Imaging, Hangzhou Medical College, Hangzhou, China; 3 Department of Emergency Medicine, Ningbo No.2 Hospital, Ningbo, China

**Keywords:** Castleman disease, Renal tumor, Acute abdominal pain, Hyaline-vascular type, Pathological examination, T-cell proliferation

## Abstract

**Introduction/Background:**

Castleman disease (CD) is a rare lymphoproliferative disorder, with primary renal involvement being exceptionally uncommon. Most cases are asymptomatic and discovered incidentally. This report describes an unusual presentation of renal CD manifesting as acute abdominal pain, highlighting the diagnostic challenges in differentiating it from renal malignancies.

**Case Presentation:**

A 55-year-old Chinese female presented to the emergency department with sudden-onset epigastric pain radiating to the left lumbar region, accompanied by nausea and vomiting. Emergency CT revealed a left renal mass measuring 80 × 75 mm with internal nodular calcifications. Contrast-enhanced CT demonstrated heterogeneous enhancement patterns suspicious for chromophobe renal cell carcinoma. Laboratory tests showed elevated transaminases and microscopic hematuria. Given the inability to exclude malignancy, the patient underwent laparoscopic left nephrectomy. Histopathological examination revealed hyaline vascular Castleman disease with characteristic “onion-skin” lymphoid follicles, atrophic germinal centers, and “lollipop-like” vascular structures. Immunohistochemistry confirmed mixed B- and T-cell proliferation without evidence of monoclonality. The patient recovered uneventfully, with complete symptom resolution.

**Conclusion:**

This case emphasizes that CD should be considered in the differential diagnosis of renal masses, particularly those with unusual enhancement patterns and calcifications. While definitive diagnosis relies on histopathology, recognition of this entity may guide appropriate surgical planning and avoid unnecessary radical procedures. The excellent prognosis following complete excision underscores the importance of accurate diagnosis.

## 1 INTRODUCTION

Castleman disease, also known as giant lymph node hyperplasia, was first reported by Benjamin Castleman in 1954. Its main characteristics include abnormal lymphoid follicular hyperplasia, hyaline vascular changes, or plasma cell infiltration. Histologically, it is classified into the hyaline-vascular, plasma cell, and mixed types. Clinical classifications include unicentric Castleman disease (UCD) and multicentric Castleman disease (MCD), with the latter further divided into idiopathic multicentric Castleman disease (iMCD) and human herpesvirus 8-associated multicentric Castleman disease (HHV8+ MCD) [1]. UCD is more common, typically affecting lymph node-rich regions and presenting as asymptomatic masses. iMCD and HHV8+ MCD may be accompanied by systemic symptoms such as fever and, in severe cases, can involve multiple organ systems. However, primary renal Castleman disease is extremely rare and typically presents with non-specific abdominal pain. Imaging may reveal well-defined renal masses that require differentiation from renal cell carcinoma, lymphoma, and other malignancies. Renal lymphoma, the most common lymphomatous involvement of abdominal organs, typically presents as hypovascular masses with homogeneous hypoenhancement on contrast-enhanced imaging and may involve multiple bilateral lesions. Clear differentiation between renal lymphoma and renal cell carcinoma is critical, as lymphoma is treated with chemotherapy, whereas RCC requires surgical intervention.

This study describes a case of a 55-year-old female with renal hyaline-vascular Castleman disease (HV-CD). The main contributions of this report include: (1) documenting a rare presentation of renal CD with acute abdominal pain, expanding the clinical spectrum of this entity; (2) providing a comprehensive analysis combining imaging features, pathological findings, and differential diagnosis; (3) conducting a systematic literature review of previously reported renal CD cases to identify common patterns and diagnostic challenges; (4) emphasizing the value of multidisciplinary collaboration and the potential role of preoperative biopsy in diagnosis. Through this case presentation, the study aims to enhance clinical awareness and optimize diagnostic and treatment strategies for this rare condition.

The manuscript begins with a detailed case report that outlines the patient’s clinical presentation, imaging findings, and treatment course. This is followed by a comprehensive discussion integrating clinical manifestations with a review of the relevant literature, along with an analysis of characteristic imaging and pathological features, established diagnostic criteria, and current treatment strategies. The paper concludes by highlighting key clinical implications and offering practical recommendations to inform diagnostic approaches.

## 2 CASE PRESENTATION

**Fig. (1):**
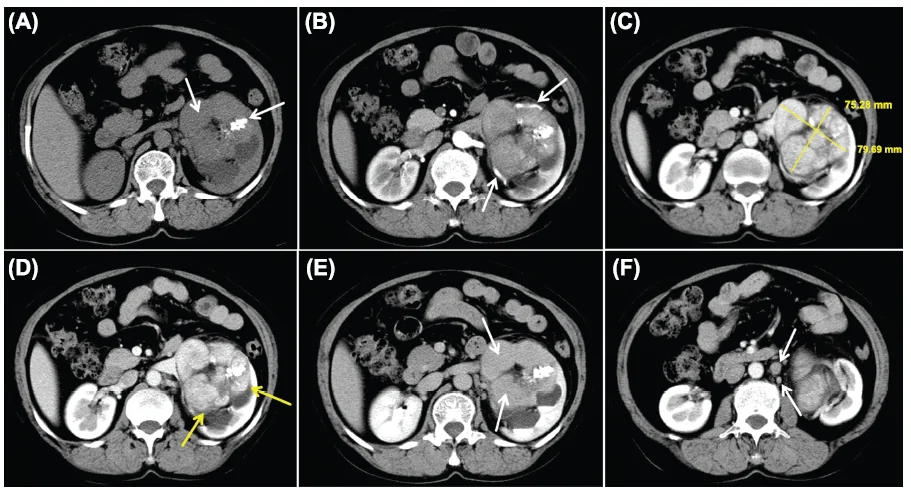
CT imaging features of left renal Castleman disease. (**A**) Non-contrast CT showing a soft tissue mass with nodular hyperdensities (white arrowheads). (**B**) Arterial phase demonstrating mild heterogeneous enhancement with displacement of the left renal artery (white arrowheads). (**C**) Venous phase showing progressive enhancement with well-defined margins measuring 80×75 mm. (**D**) Coronal reconstruction demonstrating pelvicalyceal compression with hydronephrosis (yellow arrowheads). (**E**) Delayed phase revealing contrast washout (white arrowheads). (F) Para-aortic lymphadenopathy at the level of the left renal hilum (white arrowheads).

A 55-year-old female was admitted to the emergency department on February 23, 2025, with a chief complaint of “sudden epigastric pain with nausea and vomiting for 2 hours.” The patient experienced sudden epigastric pain without obvious precipitating factors, characterized by intermittent colicky pain radiating to the left lumbar region and accompanied by nausea and vomiting of small amounts of gastric contents. She denied urinary frequency, urgency, gross hematuria, or bowel dysfunction. Her medical history included hypertension without regular medication compliance. On admission, vital signs were as follows: clear consciousness, temperature 36.5°C, respiratory rate 20 breaths/min, pulse 60 beats/min, and blood pressure 177/93 mmHg. Physical examination revealed a soft abdomen with mild epigastric tenderness without rebound tenderness, negative bilateral costovertebral angle tenderness, no tenderness along the ureteral tract, and no suprapubic dullness to percussion. Emergency non-contrast CT of the urinary system showed a left renal mass with hydronephrosis, demonstrating soft-tissue density with multiple nodular hyperdensities (Fig. **1A**). The preliminary diagnosis was a left renal mass and primary hypertension, and the patient was admitted for further evaluation.

The following day, contrast-enhanced abdominal CT was performed. The mass demonstrated mild heterogeneous enhancement in the arterial phase (Fig. **1B**), with progressive enhancement in the portal venous phase, reaching approximately 80 × 75 mm in maximum diameter (Fig. **1C**). The left renal vessels and collecting system showed displacement without evidence of vascular invasion (Fig. **1B**, **D**). Delayed-phase images revealed contrast washout (Fig. **1E**), with enhancement remaining lower than that of normal renal parenchyma in all phases. Mild hydronephrosis of the left renal pelvis was observed. Multiple enlarged lymph nodes were identified along the abdominal aorta, demonstrating mild enhancement (Fig. **1F**).

Laboratory tests showed: alanine aminotransferase (ALT) 55 U/L (normal range: 0-35 U/L), aspartate aminotransferase (AST) 97 U/L (normal range: 0-40 U/L), and white blood cells 3+ per high-power field on urinalysis (normal range: negative or 0-5 per HPF). Complete blood count, serum amylase, and cardiac enzymes were within normal limits. Based on these findings, the working diagnosis was a malignant renal mass (suspected chromophobe renal cell carcinoma) with mild left hydronephrosis, para-aortic lymphadenopathy, and abnormal liver function. After comprehensive evaluation, the patient’s liver function abnormality was deemed manageable, with no contraindications to surgery. On March 2, 2025, the patient underwent laparoscopic left nephrectomy with perinephric adhesiolysis and hilar lymph node dissection under general anesthesia, followed by postoperative antibiotic therapy, fluid supplementation, and supportive care.

However, postoperative pathology unexpectedly revealed left renal hyaline-vascular Castleman disease (HV-CD) with interstitial cell proliferation. Characteristic microscopic findings are illustrated in Fig. (**2**). Numerous hyperplastic lymphoid follicles were observed in a dense arrangement, some exhibiting an “onion-skin” appearance with atrophic germinal centers. High magnification revealed capillary proliferation within germinal centers with swollen endothelial cells, thickened vessel walls with hyaline changes, and small vessels penetrating follicular centers, forming “lollipop-like” structures. Interfollicular areas showed high endothelial venule proliferation with hyalinization and thickened vessel walls. Mantle zone lymphocytes demonstrated increased proliferation with a concentric arrangement surrounding atrophic germinal centers.

Immunohistochemistry results showed: CD20(+), CD79a(+), CD3(+), CD5(+), indicating mixed B- and T-cell proliferation; CD23(-), Bcl-6(-), CD10(-), suggesting disrupted follicular architecture; Ki-67 (15%+), indicating moderate proliferative activity; Bcl-2(+), CyclinD1(-), EBER(-), ruling out mantle cell lymphoma and EBV-associated lymphoproliferative disease; Kappa(+/-), Lambda(+/-), showing no evidence of monoclonal plasma cell proliferation. Given the absence of evidence of involvement at other sites, multicentric Castleman disease was excluded, and the patient was definitively diagnosed with unicentric renal Castleman disease.

The patient’s postoperative recovery was uneventful, with satisfactory wound healing and complete resolution of symptoms. Physical examination prior to discharge showed clear consciousness, a soft abdomen, no costovertebral angle tenderness, and no tenderness along the ureteral tract. The patient was discharged on March 15, 2025, with recommendations for regular follow-up monitoring of renal function and liver function. A standardized surveillance protocol was implemented, incorporating serial renal function monitoring and cross-sectional imaging at 6-month intervals. During regular follow-up visits, the patient reported no significant abnormalities.

## 3 DISCUSSION

### 3.1 Clinical Manifestations and Literature Review

Renal Castleman disease lacks specific clinical manifestations. A comprehensive literature review identified 10 previously reported cases of renal Castleman disease [2-11] (Table **1**). Most cases were from Asia, with an average patient age of approximately 50 years and no significant gender differences. Lesions were predominantly located in the renal sinus, with unicentric Castleman disease (UCD) and hyaline-vascular type being the most common. Notably, all reported cases presented with non-specific symptoms.

Most patients present with asymptomatic renal masses [2-5], while a minority present with abdominal pain [6], lumbar pain [7], or hematuria. Very few patients have associated comorbidities. Multicentric Castleman disease (MCD) involving the kidneys may present with systemic symptoms such as fever, weight loss, and anemia [8,9]. These non-specific clinical presentations pose significant challenges for the diagnosis of renal CD.

The present case is particularly noteworthy for its presentation with acute abdominal pain-a symptom reported in only one previous case [6]. This clinical presentation initially suggested acute surgical pathology such as renal colic or acute abdomen, rather than a lymphoproliferative disorder. Compared with the cases in Table **1**, this patient’s presentation underscores the diagnostic challenge when renal CD mimics acute urological emergencies. The sudden onset of epigastric pain radiating to the left lumbar region, accompanied by nausea and vomiting, necessitated emergency evaluation and ultimately led to the discovery of the renal mass. This atypical presentation emphasizes the importance of including CD in the differential diagnosis of acute abdominal pain associated with renal masses.

### 3.2 Imaging Features

Given these diagnostic challenges, imaging characterization plays a crucial role in the diagnostic pathway. A previous report [12] found that CT plain scans show lesions with slightly higher density relative to renal parenchyma, whereas other hypervascular tumors typically demonstrate lower density-a feature reflecting the relationship between the lesion and lymphocyte composition. However, most reports describe imaging features only as well-defined or mildly enhancing [4-7,11] soft tissue masses, with limited analysis of internal mass characteristics (such as density or composition) that might have differential diagnostic value.

In the present case, contrast-enhanced CT demonstrated progressive enhancement with a washout pattern and associated lymphadenopathy. Specifically, the mass showed mild heterogeneous enhancement in the arterial phase, progressive enhancement in the portal venous phase, and contrast washout in the delayed phase, with enhancement remaining lower than that of normal renal parenchyma in all phases. These features, combined with the well-defined margins and para-aortic lymphadenopathy, while not specific for Castleman disease, raised suspicion for malignancy including chromophobe renal cell carcinoma or lymphoma. The presence of multiple enlarged lymph nodes along the abdominal aorta is an important imaging finding that, in retrospect, should prompt consideration of lymphoproliferative disorders including CD.

The imaging characteristics observed in this case align with several previously reported cases showing mildly enhancing masses with well-defined margins [5-7]. However, the progressive enhancement pattern observed here has been less commonly described, highlighting the variable imaging presentations of renal CD. This variability underscores the difficulty in establishing specific imaging criteria for preoperative diagnosis.

### 3.3 Pathological Examination and Diagnostic Criteria

Pathological examination remains the gold standard for definitive diagnosis of Castleman disease. However, the defining criteria for the three histological subtypes-hyaline-vascular, plasma cell, and mixed types-remain unclear, lacking distinct diagnostic boundaries [13]. Some studies suggest that all patients tend to have increased vascular distribution and at least some atrophic germinal centers [14].

The hyaline-vascular type, as observed in the present case, primarily demonstrates germinal center degeneration, vascular proliferation, and hyalinization. The plasma cell type presents with follicular hyperplasia and interfollicular mature plasmacytosis, while the mixed type exhibits characteristics of both. Key pathological features include “onion-skin” lymphoid follicular structures, “lollipop-like” vascular penetration of follicles, and hyaline changes in capillaries-all of which were present in this case.

Microscopic examination of the present case revealed numerous hyperplastic lymphoid follicles in a dense arrangement, some exhibiting the characteristic “onion-skin” appearance with atrophic germinal centers. High magnification showed capillary proliferation within germinal centers with swollen endothelial cells, thickened vessel walls with hyaline changes, and small vessels penetrating follicular centers, forming the pathognomonic “lollipop-like” structures. Interfollicular areas demonstrated high endothelial venule proliferation with hyalinization and thickened vessel walls. These findings, combined with an immunohistochemical profile showing mixed B- and T-cell proliferation without evidence of monoclonality, confirmed the diagnosis of hyaline-vascular type Castleman disease.

The immunohistochemical panel in this case was essential for excluding other differential diagnoses. CD20(+) and CD79a(+) confirmed B-cell lineage, while CD3(+) and CD5(+) identified T cells. The negative staining for CD23, Bcl-6, and CD10 indicated disrupted follicular architecture, distinguishing this from reactive follicular hyperplasia. The moderate Ki-67 proliferation index (15%) and the absence of Bcl-2 and CyclinD1 expression helped exclude mantle cell lymphoma. Negative EBER staining ruled out EBV-associated lymphoproliferative disease, while the polyclonal pattern of Kappa and Lambda light chains excluded plasma cell neoplasms.

### 3.4 Treatment and the Role of Preoperative Biopsy

Treatment strategies for renal CD do not differ significantly from CD at other sites, with individualized approaches primarily based on clinical subtype. Complete surgical resection is the optimal treatment for UCD, offering high long-term survival rates and low recurrence rates [15]. The extent of surgery should be determined based on preoperative assessment.

A critical clinical consideration highlighted by the present case is the potential role of preoperative biopsy in the diagnostic pathway. In this case, preoperative imaging suggested malignancy, leading to radical nephrectomy. However, had UCD been confirmed preoperatively through percutaneous core needle biopsy, nephron-sparing surgery might have been considered, potentially preserving renal function. The decision to pursue preoperative biopsy for suspicious renal masses remains controversial in urological practice. Arguments supporting biopsy include the potential to avoid unnecessary radical surgery for benign lesions, particularly in patients with solitary kidneys or compromised renal function. However, concerns include the risk of tumor seeding, bleeding complications, and the possibility of non-diagnostic sampling due to the heterogeneous nature of some renal masses.

For masses with imaging features atypical for conventional renal cell carcinoma-such as well-defined margins, progressive enhancement pattern, and associated lymphadenopathy without clear vascular invasion-multidisciplinary discussion should consider the potential benefits of preoperative tissue diagnosis. In the context of suspected CD, where characteristic histopathological features are required for definitive diagnosis and where the lesion is benign, preoperative biopsy could fundamentally alter surgical planning and preserve renal parenchyma. This case underscores the importance of refining diagnostic algorithms to balance the goals of definitive diagnosis, organ preservation, and oncological safety. For unresectable UCD, conservative treatment may be adopted. Research indicates that radiotherapy is an acceptable alternative for unresectable UCD but should be avoided when possible in younger patients [16].

### 3.5 Pathogenesis and Epidemiology

The pathogenesis of Castleman disease has not been fully elucidated. Current research suggests that different subtypes have distinct pathogenic mechanisms. In UCD, follicular dendritic cells may exhibit clonal tumor characteristics [13], and abnormal secretion of IL-6 in the local microenvironment can promote immune cell proliferation and activation, leading to characteristic lymphoid tissue pathological changes [17]. The core pathogenic mechanism of MCD is an IL-6-driven cytokine storm [18]. In the HHV8+ MCD subtype, viral-encoded enhanced viral IL-6 plays a pathogenic role, while simultaneously encoding v-FLIP and miRNA-K1 proteins that continuously activate the NF-κB signaling pathway and upregulate angiogenic factors such as VEGF [19], collectively promoting B-cell and plasma cell abnormal proliferation, angiogenesis, and systemic inflammatory responses.

As a group of rare diseases with clinical and histopathological heterogeneity, current epidemiological research on Castleman disease remains limited. One study developed an algorithm to identify potential CD patients, estimating an annual incidence of 21-25 per million [20]. UCD, as the most common clinical subtype, has an annual incidence of 16 per million [2]. Another study estimated 30,000-100,000 UCD cases in the United States [21], with an average age of onset of 35 years and a slightly higher incidence in females. Additionally, UCD patients are typically diagnosed 20 years earlier than MCD patients, although patients of all age groups can develop any form of CD [13]. However, reliable incidence data specifically for primary renal CD are currently lacking. Due to high misdiagnosis rates, insufficient pathological biopsy implementation, and variations in clinician awareness, the epidemiological characteristics of this entity require further investigation.

## CONCLUSION

Renal Castleman disease has an extremely low incidence worldwide, but clinicians should remain vigilant and include it in the differential diagnosis of patients with acute abdominal pain and renal masses. Its non-specific signs overlap with common diseases such as renal cell carcinoma and lymphoma, potentially leading to misdiagnosis. Imaging likewise lacks pathognomonic features; when suspected, contrast-enhanced examination may provide valuable diagnostic clues. Pathological examination is currently the gold standard for definitive diagnosis, with particular attention to CD-characteristic “onion-skin” lymphoid follicular structures and hyaline changes in capillaries. Notably, UCD, the most common clinical subtype, is predominantly a benign lesion; therefore, accurate pathological diagnosis is essential for guiding appropriate therapeutic decisions, potentially avoiding unnecessary radical surgery. Establishing a multidisciplinary consultation system involving emergency medicine, surgery, radiology, and pathology departments, along with a rational diagnostic workflow, is crucial. For suspected cases, timely multidisciplinary consultation should be conducted to obtain pathological evidence as early as possible, aiming to achieve early, precise diagnosis and rational intervention to improve patient prognosis.

## Figures and Tables

**Fig. (2):**
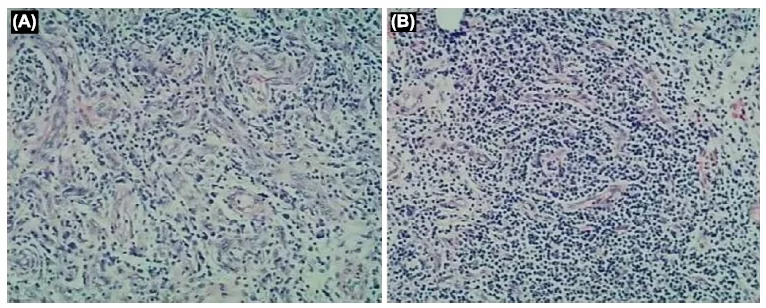
Microscopic features of hyaline-vascular Castleman disease (H&E stain). (**A**) Germinal center showing capillary proliferation with swollen endothelial cells, thickened vessel walls with hyaline changes, and “lollipop-like” vascular penetration of follicular centers. (**B**) Interfollicular areas demonstrating high endothelial venule proliferation with hyalinization and thickened vessel walls.

**Table 1:** Imaging and pathological features of previously reported cases of renal Castleman disease.

**Study**	**Region**	**Age (years)**	**Gender**	**Symptoms**	**Location/Size (mm)**	**Imaging Features**	**Clinical Type**	**Pathological Type**	**Para-aortic Lymphadenopathy**
Zhang (2022) [2]	Asia	40	F	Asymptomatic	Left renal sinus 47 × 40 × 38	CT: Ill-defined soft tissue mass with marked heterogeneous enhancement	UCD	Hyaline vascular CD	(+)
Yaynishet (2024) [3]	Africa	42	M	Asymptomatic	Right renal hilum 43 × 38	MRI: Intermediate signal intensity on both T1 and T2 sequences	UCD	Hyaline vascular CD	(-)
Li (2019) [4]	Asia	56	M	Asymptomatic	Right middle-upper kidney 45 × 40 × 20	CT: Isodense solitary soft tissue mass	UCD	Hyaline vascular CD	(-)
Guo X (2019) [5]	Asia	65	F	Asymptomatic	Right lower renal sinus	CT: Mildly enhanced soft tissue mass; MRI: Low signal on T2, high signal on DWI	UCD	Plasma cell CD	(-)
Radfar H (2015) [6]	Asia	32	F	Left abdominal pain	Left renal hilum 70 × 70	CT: Well-defined, homogeneously enhancing soft tissue mass without calcification or necrosis	UCD	/	(-)
Fu (2023) [7]	Asia	46	F	Left lumbar pain	Left renal pelvis 37 × 35× 35	CT: Solitary soft tissue mass; moderate enhancement at base in corticomedullary phase, heterogeneous enhancement in nephrographic phase, reduced enhancement in excretory phase	UCD	Hyaline vascular CD	(+)
Ueda (2024) [8]	Asia	51	M	Anemia, weight loss, rash, and fever	Bilateral renal pelvis	CT: Soft tissue masses surrounding the bilateral renal pelvis	MCD	/	Generalized (+)
Sawada (2024) [9]	Asia	55	M	High fever, diarrhea	Bilateral renal interstitial infiltration	CT: Lymphadenopathy in axillary, hilar, mediastinal, para-aortic, and inguinal regions	MCD	/	Generalized (+)
Kim (2012) [10]	Asia	59	M	Exertional dyspnea (CD with secondary cardiac tamponade)	Left renal sinus, left mid-renal parenchyma	CT: Mildly enhanced soft tissue mass, moderate pericardial effusion; MRI: Renal sinus mass with intermediate signal on T1, low signal on T2; renal parenchymal mass isointense on both T1 and T2	UCD	Plasma cell CD	(-)
Chen (2022) [11]	Asia	56	F	Oliguria, limb edema (renal failure with CD)	Right lower renal parenchyma 13 × 13 × 11	CT: Solitary soft tissue mass	UCD	Hyaline vascular CD	(-)

**Abbreviations**: F, female; M, male; CD, Castleman disease; UCD, unicentric Castleman disease; MCD, multicentric Castleman disease.

## Data Availability

The original contributions supporting this study are available in the article. Further queries should be directed to the corresponding author [Q.D].

## LIST OFABBREVIATIONS

CD: Castleman disease
CT: Computed tomography
HV: Hyaline-vascular
MCD: Multicentric Castleman disease
PC: Plasma cell
UCD: Unicentric Castleman disease

## STANDARDS FOR REPORTING

CARE guidelines were followed.
